# Supplementary material for: Costs of Providing HIV Self-Test Kits to Pregnant Women Living with HIV for Secondary Distribution to Male Partners in Uganda
Source: Diagnostics (Basel). 2020 May 19;10(5):318. doi: 10.3390/diagnostics10050318 (PMC7277977; doi:10.3390/diagnostics10050318)
Supplement: Supplementary file 1 [file diagnostics-10-00318-s001.pdf]

Article

Costs of Providing HIV Self-Test Kits to Pregnant Women Living With HIV for Secondary Distribution to Male Partners in Uganda

Michelle A. Bulterys [1], Andrew Mujugira [1,2], Agnes Nakyanzi [2], Miriam Nampala [2], Geoffrey Taasi [3], Connie Celum [1] and Monisha Sharma [1,*]

[1] International Clinical Research Center, Department of Global Health, University of Washington, Seattle, WA, 98104, USA; mbult@uw.edu (M.A.B.); mujugira@uw.edu (A.M.); ccelum@uw.edu (C.C.)
[2] Infectious Diseases Institute, Makerere University, Kampala, P.O. Box 22418, Uganda; anakyanzi@idi.co.ug (A.N.); mnampala@idi.co.ug (M.N.)
[3] Division of HIV Testing, Uganda Ministry of Health, Kampala, P.O. Box 7272, Uganda; taasi.taasi@gmail.com
* Correspondence: msharma1@uw.edu

Supplementary Materials

Methods:

Table 1. Activities, inputs, and data sources.

Activities	Resources/inputs	Source
Start-up		
Materials development: SOPs, policy guidelines, training materials	Personnel time, supplies, consultancies	Expense reports; staff interviews
Stakeholder meetings	Personnel time, venue, refreshments, per diem, travel, supplies	Expense reports; staff interviews
Trainings of program staff	Personnel time, venue, refreshments, per diem, travel, supplies	Expense reports; staff interviews
M&E start-up (if any)	Personnel time, supplies, consultancies, contracted services	Expense reports; staff interviews
Recurrent		
Personnel time of health workers	Facility-level data (Personnel time, per diem, travel, phone/ internet, office space) MoH data for scenario	Clinic expense reports; staff interviews; time motion; MoH pay scales and interviews
Personnel time of management/supervision	Personnel time, per diem, travel, phone/internet, office space	Expense reports; staff interviews; time motion

Capital costs (transport, IT equipment for data abstraction)	Personnel time, vehicles, supplies, fuel, IT equipment)	Expense reports; staff interviews
Intervention (HIVST kits)	Cost quantity of HIVST kits purchased in bulk	Study budget
HIV rapid testing	Personnel time, cost of rapid tests	Expense reports; staff interviews; Time and motion

Scenario 1: As-Studied

In the As-Studied scenario, we assumed five nurses work 264 days of the year. We estimated the number of women that could be reached if the nurses worked at full capacity, giving their full time to delivering this intervention. Based on TM observations and staff interviews, we estimated that it takes an average of 50 minutes to train and counsel a PWLHIV on how to deliver an HIVST kit to her male partner. Assuming each nurse worked 6 hours a day (accounting for distractions and delays), a nurse could reach an average of 7 women per day. Five nurses working 264 days a year, reaching 7 women per day, would equate to 9,240 women reached in one year. The same process was used to estimate the number of men who could be linked to clinic testing. The only difference comes from the amount of time it takes to see one male; we estimate it takes 40 minutes to see an HIV-negative male and 45 minutes to see an HIV-positive male. Annually, we estimate 11,880 HIV-negative men can be seen and 10,560 HIV-positive men can be seen if the clinic is working at full capacity. Costs were annualized over 5 years assuming a 3% discount rate. The column titled "% of intervention" in some costing tables indicates the proportion of that cost that was spent on delivering this intervention, as opposed to other interventions or routine care.

Scenario 1: Summary Tables of Cost Metrics

Cost of distributing HIVST kits to HIV+ pregnant women

Cost category	Total USD	Category cost/woman
Personnel	$87,598.17	$9.48
Vehicles + fuel + maintenance	$278.53	$0.03
Office supplies	$4,072.38	$0.44
Utilities, building, overhead	$7,197.95	$0.78
Other equipment	$32.87	$0.00
Startup training costs	$1,663.22	$0.18
HIVST kits ($2 each)	$18,480.00	$2.00
**Total cost for one woman**	**$119,323.11**	**$12.91**

Cost of linking a HIV+ male partner to the clinic for HIV testing

Cost category	USD	Cost/man
Personnel	$87,598.17	$8.30
Vehicles + fuel + maintenance	$278.53	$0.03

Office supplies	$4,072.38	$0.39
Utilities	$7,197.95	$0.68
Other equipment	$32.87	$0.00
Startup training costs	$1,663.22	$0.16
Male reimbursement	$85,852.80	$8.13
Clinical supplies for rapid HIV testing	$24,606.58	$2.33
**Total cost of testing HIV+ male at clinic**	**$211,302.49**	**$20.02**

Cost of linking a HIV- male partner to the clinic for HIV testing

Cost category	USD	Cost/man
Personnel	$87,598.17	$7.37
Vehicles + fuel + maintenance	$278.53	$0.02
Office Supplies	$4,072.38	$0.34
Utilities	$7,197.95	$0.61
Other equipment	$32.87	$0.00
Startup training costs	$1,663.22	$0.14
Male reimbursement	$96,584.40	$8.13
Clinical supplies for rapid HIV testing	$24,606.58	$2.07
**Total cost of testing HIV- male at clinic**	**$222,034.09**	**$18.68**

Scenario 1: Personnel costs (excluding research time)

Position Title	Number	Gross Annual Salary	Total Cost	% for Intervention	Total for Intervention
Physician/Investigator	1	32,640	32,640	20%	6,528
Pharmacy Technician	1	10,667	14,376	40%	5,750
Data officer (assume lower in MOH scenario)	1	5,051	6,807	20%	1,361
Team lead nurse	1	8,760	11,806	100%	11,806
Administrator (finance manager)	1	10,537	14,201	20%	2,840
Data Manager	1	9,851	13,276	20%	2,655
Lab Technician	1	8,998	12,127	20%	2,425
Peer Counselor Mothers (Study stipend only)	2	2,026	4,052	100%	4,052
Recruitment Officer	1	650	876	100%	876
Nurses	4	7,764	41,854	100%	41,854
Facilities Assistant	1	11,055	14,899	50%	7,449
**Total Staff:**	**21**				**87,598**

Scenario 1: Clinical Supplies (only needed for male testing)

We excluded all standard of care costs, meaning no additional clinical supplies were needed for women receiving kits, which were free of charge at the clinic. The cost of clinical supplies needed for testing men included gloves, rapid HIV test and syphilis test.

Supplies needed for 1 Male Enrollment Visit just HIV testing	Cost/unit ($)	Number units	Total cost
HIV determine testing kits (100/unit)	1.03	1	1.03
Gloves (100/unit)	0.51	2	1.03
Syphilis rapid testing kits (50/unit)	0.27	1	0.27
**Total cost of clinical supplies:**			**2.33**

Scenario 1: Vehicles, fuel and maintenance

Item	Unit Cost	Number	Total Cost	Useful Life (yrs)	Annual Cost	% for Intervention	Total for Intervention
4WD Vehicle	9,483	1	9,483	5	2,091	5%	105
Motorcycle	271	1	271	5	60	5%	3
				**Total cost for vehicles**			**108**

Item	Number	Unit Cost	Total Cost	% for Intervention	Total for Intervention
Motorcycle fuel	12	5.8	70	5%	3
Motor vehicle fuel	12	60	716	5%	36
Insurance of motorcycle	1	5	5	5%	0
Maintenance for motorcycle	12	27	325	5%	16
Maintenance for motor vehicle	4	542	2,168	5%	108
Insurance of motor vehicle	1	135	135	5%	7
		**Total cost for fuel and maintenance**			**171**

Scenario 1: Office supplies

Item	Number	Unit Cost	Total Cost	% for Intervention	Total for Intervention
Participant appointment cards	500	300	41	100%	41
Storage cabinets	7	700,000	1,328	100%	1,328
Extension cable	4	55,000	60	100%	60
Participant binder files	500	13,000	1,761	50%	881
Photocopying paper	300	25,000	2,032	50%	1,016
Plastic files	500	3,000	406	50%	203
Toner cartridge	4	700,000	759	50%	379
Counter books	12	10,000	33	50%	16
Suspension files	500	2,200	298	50%	149
		**Total cost for office supplies**			**4,072**

Scenario 1: Utilities, building and overhead

Item	Number	Unit Cost	Total Cost	% for Intervention	Total for Intervention
Airtime; Credit (per month)	12	19	228	100%	228
Electricity (per month)	12	135.5	1,626	100%	1,626
Building/infrastructure maintenance	1	5,000	5,000	100%	5,000
Internet (for 3 Mifi devices per month)	12	89	1,073	20%	215
Garbage disposal	4	32.5	130	100%	130
	**Total cost for utilities and building**				**7,198**

Scenario 1: Start up and refresher trainings

Item	Number	Unit Cost	Total Cost	% for Intervention	Total for Intervention
Provider training	1	163	163	50%	81
Refresher training	2	434	217	50%	108
Training materials	1	53	53	50%	27
	**Total administrative training costs**				**216**

Item	# days	# hours	Number	Annual Salary	Hourly salary	Time costs
Team lead nurse	3 days	21	1	11,806	7.45	156.52

Nurses	3 days	21	4	41,854	26.42	554.88
Coordinator to conduct training	3 days	21	1	32,640	20.61	432.73
Data manager	3 days	21	1	9,851	6.22	130.60
Peer support mothers	3 days	21	2	4,000	2.53	53.03
Lab technician	3 days	21	1	8,998	5.68	119.29
	**Total costs for personnel training time**					**1447.05**

Total training costs = $216 + $1447 = $1,663

Scenario 2: MOH Implementation

In the Study in MOH scenario, we assumed the same numbers of women and men reached by five nurses working at full capacity for 264 days of the year.

Scenario 2: Summary tables of cost metrics

**Cost of distributing HIVST kits to HIV+ pregnant women**		
**Cost category**	**Total USD**	**Cost/woman**
Personnel	$46,731.41	$5.06
Vehicles + fuel + maintenance	$278.53	$0.03
Office supplies	$4,072.38	$0.44
Utilities, building, overhead	$7,197.95	$0.78
Other equipment	$32.87	$0.00
Startup training costs	$791.19	$0.09
HIVST kits ($2 each)	$18,480.00	$2.00
**TOTAL COST**	**$77,584.33**	**$8.40**

Cost of linking a HIV+ male partner to the clinic for HIV testing

**Cost category**	**USD**	**Cost/man**
Personnel	$46,731.41	$4.43
Vehicles + fuel + maintenance	$278.53	$0.03
Office supplies	$4,072.38	$0.39
Utilities	$7,197.95	$0.68
Other equipment	$32.87	$0.00
Startup training costs	$791.19	$0.07
Male reimbursement	$85,852.80	$8.13
Clinical supplies for rapid HIV testing	$23,971.20	$2.27
**Total cost of testing HIV+ male at clinic**	**$168,928.33**	**$16.00**

Cost of linking a HIV- male partner to the clinic for HIV testing		
Cost category	**USD**	**Cost/man**
**Personnel**	$46,731.41	$3.93
**Vehicles + fuel + maintenance**	$278.53	$0.02
**Office supplies**	$4,072.38	$0.34
**Utilities**	$7,197.95	$0.61
**Other equipment**	$32.87	$0.00
**Startup training costs**	$791.19	$0.07
**Male reimbursement**	$96,584.40	$8.13
**Clinical supplies for rapid HIV testing**	$23,971.20	$2.02
Total cost of testing HIV- male at clinic	**$179,659.93**	**$15.12**

Scenario 2: Personnel costs

Personnel staffing structure remained the same as Scenario 1, however the annual salaries were modified to match the Uganda Ministry of Health pay scales.[1]

Position Title	Number	Gross Annual Salary	Total Cost (+34.7% benefits)	% for Intervention	Total for Intervention
Physician/Investigator	1	12,193	16,432	20%	3,286
Pharmacy Technician	1	10,667	14,376	40%	5,750
Data manager	1	9,754	13,146	20%	2,629
Team lead nurse	1	3,902	5,258	100%	5,258
Administrator (finance manager)	1	10,537	14,201	20%	2,840
Data Manager	1	9,851	13,276	20%	2,655
Lab Technician	1	1,020	1,375	20%	275
Peer Counselor Mothers (gov't salary only)	2	406	813	100%	813
Recruitment Officer	1	650	876	100%	876
Nurses (assume U6 level)	4	2,764	14,898	100%	14,898
Facilities Assistant	1	11,055	14,899	50%	7,449
**Total Staff**	**21**		**Total personnel costs**		**46,731**

Scenario 2: Clinical supplies

We excluded all standard of care costs, meaning no additional clinical supplies were needed for the female study participants. The cost of clinical supplies needed for testing men included gloves and a DUAL HIV/syphilis test.

Supplies needed for 1 Male Enrollment Visit just HIV testing	Cost/unit ($)	Number units	Total cost
Gloves (100/unit)<br>- A box of 100 gloves costs UGX 50,000 (approx $0.135/glove)	0.135	2	0.27
Syphilis rapid testing kits (50/unit)	2.000	1	2.00
**Total cost of clinical supplies:**			**2.27**

Scenario 2: Vehicles, fuel and maintenance, office supplies, utilities and building, were assumed to be the same costs as Scenario 1.

Scenario 2: Start up and refresher trainings

Item	Number	Unit Cost	Total Cost	% for Intervention	Total for Intervention
Provider training	1	163	163	50%	81
Refresher training	2	434	217	50%	108
Training materials	1	53	53	50%	27
		**Total administrative training costs**			**216**

Item	# days	# hours	Number	Annual Salary	Hourly salary	Time costs
Team lead nurse	3 days	21	1	5,258	3.32	69.71
Nurses	3 days	21	4	3,725	2.35	49.38
Coordinator (responsible for training)	3 days	21	1	16,432	10.37	217.85
Data manager	3 days	21	1	13,146	8.30	174.28
Peer support mothers	3 days	21	2	813	0.51	10.78
**Lab technician**	3 days	21	1	4,000	2.53	53.03
		**Total costs for personnel training time**				**575.03**

Total training costs = $216 + $575 = $791

Scenario 3: MOH Roll-Out

In the MOH Roll-Out scenario, we assumed two nurses would be working at full capacity for 264 days of the year. Costs were calculated from the MOH payer perspective, and therefore costs for HIVST kits were assumed to be purchased in bulk with subsidized discounts. In this scenario, HIVST is delivered by group counseling through antenatal waiting room discussions to pregnant women regardless of HIV status, instead of individual counseling for PWLHIV only (as in Scenarios 1 & 2). Total HIVST group counseling and distribution takes an average of 70 mins, with an average of 30 pregnant women attending. If nurses are working at full capacity, we assume that 150 women can receive the HIVST training in 1 day from a total of 5 trainings; however, approximately 80% of those women choose to take a kit home to deliver to their male partner. Therefore, we will assume 24 (80% of 30) women are reached in each group counseling session, equating to 120 women in a day and 63,360 in a year. In this scenario, only men who self-test positive are encouraged to link to clinic testing. Assuming that it takes an average of 24 minutes to test and counsel an HIV+ male, 15 men can be seen per day by each nurse, equating to 7920 men seen in a year.

Scenario 3: Summary tables for cost metrics

Cost of distributing an HIVST kits to HIV+ Pregnant women

Cost category	Total USD	Cost/woman
Personnel	$21,731.88	$0.34
Vehicles + fuel + maintenance	$278.53	$0.00
Office supplies	$4,072.38	$0.06
Utilities, building, overhead	$7,197.95	$0.11
Other equipment	$32.87	$0.00
Startup training costs	$411.44	$0.01
Toll free call center	$8,019.69	$0.13
HIVST kits ($2 each)	$126,720.00	$2.00
**TOTAL COST**	**$168,464.73**	**$2.65**

Cost of linking a HIV+ male partner to the clinic for HIV testing

Cost category	USD	Cost/man
Personnel	$21,731.88	$2.74
Vehicles + fuel + maintenance	$278.53	$0.04
Office supplies	$4,072.38	$0.51
Utilities	$7,197.95	$0.91
Other equipment	$32.87	$0.00
Startup training costs	$411.44	$0.05
Clinical supplies for rapid HIV testing	$17,978.40	$2.27
Toll free call center	$1,002.46	$0.13
**Total cost of testing HIV+ male at clinic**	**$52,705.90**	**$6.65**

Scenario 3: Personnel costs

Position Title	Number	Gross Annual Salary	Total Cost	% for Intervention	Total for Intervention
Senior Program Director (MOH person to supervise)	1	21,600	21,600	40%	8,640
Data manager	1	9,754	13,146	20%	2,629
Team lead nurse	1	3,902	5,258	5%	263
Peer Counselors/ Peer Mothers (gov't salary only)	4	406	1,626	100%	1,626
Recruitment Officer	1	650	876	100%	876
Nurses (assume U6 level)	1	3,691	4,974	5%	249
Facilities Assistant	1	11,055	14,899	50%	7,449
Total Staff	**10**	**Total personnel costs**			**21,732**

Scenario 3: Clinical supplies

We excluded all standard of care costs, meaning no additional clinical supplies were needed for the female study participants. The cost of clinical supplies needed for testing men included gloves and a DUAL HIV/syphilis test.

Supplies needed for 1 Male Enrollment Visit just HIV testing	Cost/unit ($)	Number units	Total cost
Gloves (100/unit)<br>- A box of 100 gloves in UG costs 50,000 (approx $0.135/glove)	0.135	2	0.27
Syphilis rapid testing kits (50/unit)	2.000	1	2.00
**Total cost of clinical supplies:**			**2.27**

Scenario 3: Vehicles, fuel and maintenance, office supplies, utilities and building, were assumed to be the same costs as Scenario 1 and 2.

Scenario 3: MOH start up and refresher trainings

Item	Number	Unit Cost	Total Cost	% for Intervention	Total for Intervention
Provider training	1	163	163	50%	81
Refresher training	2	434	217	50%	108
Training materials	1	53	53	50%	27
		**Total administrative training costs**			**216**

Item	# days	# hours	Number	Total annual salaries	Total hourly salaries	Time costs
Team lead nurse	1 days	7	1	5,258	3.32	23.24
Nurse	1 days	7	1	3,725	2.35	16.46
Coordinator (responsible for training)	1 days	7	1	16,432	10.37	72.62
Data manager	1 days	7	1	13,146	8.30	58.09
Peer support counsellors/mothers	1 days	7	4	1,626	1.03	7.19
Lab technician	1 days	7	1	4,000	2.53	17.68
		**Total costs for personnel training time**				**195.28**

Total training costs = $216 + $195 = $411

Scenario 3: Toll-Free Call Center

The Uganda Ministry of Health offers a nationwide toll-free call number (funded by PEPFAR) for any Ugandan interested in learning more about or being counseling through HIVST. The call center phone number is printed on all HIVST kits provided in Uganda. Phone counselors are trained and callers can request to speak to a male or female counselor. All call center costs were provided directly by the Uganda Ministry of Health and we assumed that these were annualized at 5 years with 3% discount. Assuming that there are 1,665,000 live births annually in Uganda, and 50% of pregnant women take home HIVST kits (832,500). In Scenario 3, we assumed 63,360 women would be reached through our hypothetical clinic implementation (see description about Scenario 3 above), which is 8% of the 832,500 women assumed to take home an HIVST. We calculated 8% of the total annual call center costs ($105,372) which was $8,019.69. This total cost divided by the total number of women reached in our hypothetical scenario (63,360), we assume the call center costs $0.13 for every kit taken home.

Item	Number	Unit Cost	Total in USD	Total for Intervention
Airtime for inbound and outbound calls	12	1354.75	16,257	16,257
Customer relations manager	1	27,095.00	27,095	27,095
Call center system maintenance	12	270.92	3,251	3,251
Human resources	24	758.67	18,208	18,208
Website	1	813.00	813	813
smart phones	2	217.00	434	95
Laptop	1	1,219.00	1,219	266
Headsets	4	325.25	1,301	284
Media	6	541.80	3,251	3,251
advertisements	20	54.20	1,084	1,084

print advert	40,000	0.04	1,626	1,626
SMS platform (reminders)	5000	0.65	3,251	3,251
Promotional materials	1	18,154.00	18,154	18,154
Call center marketing in facilities	24	489.08	11,738	11,738
**TOTAL COST**				**105,372**

References

1. Uganda Ministry of Public Service. *Salary Structure FY 2018/2019 (Schedule 1-12). https://publicservice.go.ug/download/salary-structure-fy-2018-2019-schedule-1-12/. Accessed on March 1, 2020.*
